# Digital expression profiling of novel diatom transcripts provides insight into their biological functions

**DOI:** 10.1186/gb-2010-11-8-r85

**Published:** 2010-08-25

**Authors:** Uma Maheswari, Kamel Jabbari, Jean-Louis Petit, Betina M Porcel, Andrew E Allen, Jean-Paul Cadoret, Alessandra De Martino, Marc Heijde, Raymond Kaas, Julie La Roche, Pascal J Lopez, Véronique Martin-Jézéquel, Agnès Meichenin, Thomas Mock, Micaela Schnitzler Parker, Assaf Vardi, E Virginia Armbrust, Jean Weissenbach, Michaël Katinka, Chris Bowler

**Affiliations:** 1 Institut de Biologie de l'Ecole Normale Supérieure, CNRS UMR 8197 INSERM U1024, Ecole Normale Supérieure, 46 rue d'Ulm, 75005 Paris, France; 2 Current address: EMBL - European Bioinformatics Institute, Wellcome Trust Genome Campus, Hinxton, Cambridge CB10 1SD, UK; 3 CEA - Institut de Génomique, Genoscope and CNRS UMR 8030, 2 rue Gaston Crémieux CP5706, 91057 Evry, France; 4 Current address: J Craig Venter Institute, 11149 N. Torrey Pines Rd, Suite 220, La Jolla, CA 92037, USA; 5 Physiologie et Biotechnologie des Algues, IFREMER, BP 21105, 44311 Nantes, France; 6 Marine Biogeochemistry, IFM-GEOMAR Leibniz-Institut für Meereswissenschaften, Düsternbrooker Weg 20, D-24105 Kiel, Germany; 7 Université de Nantes, EA 2160, Laboratoire 'Mer, Molécule, Santé', Faculté des Sciences et Techniques, 2 rue de la Houssinière, 44322, BP 92208, 44322 Nantes Cedex 3, France; 8 School of Oceanography, University of Washington, 616 NE Northlake Place, Seattle, WA 98105, USA; 9 University of East Anglia, School of Environmental Sciences, Norwich Research Park, Norwich NR4 7TJ, UK; 10 Department of Plant Sciences, Weizmann Institute of Science, Rehovot 76100, Israel

## Abstract

**Background:**

Diatoms represent the predominant group of eukaryotic phytoplankton in the oceans and are responsible for around 20% of global photosynthesis. Two whole genome sequences are now available. Notwithstanding, our knowledge of diatom biology remains limited because only around half of their genes can be ascribed a function based onhomology-based methods. High throughput tools are needed, therefore, to associate functions with diatom-specific genes.

**Results:**

We have performed a systematic analysis of 130,000 ESTs derived from *Phaeodactylum tricornutum *cells grown in 16 different conditions. These include different sources of nitrogen, different concentrations of carbon dioxide, silicate and iron, and abiotic stresses such as low temperature and low salinity. Based on unbiased statistical methods, we have catalogued transcripts with similar expression profiles and identified transcripts differentially expressed in response to specific treatments. Functional annotation of these transcripts provides insights into expression patterns of genes involved in various metabolic and regulatory pathways and into the roles of novel genes with unknown functions. Specific growth conditions could be associated with enhanced gene diversity, known gene product functions, and over-representation of novel transcripts. Comparative analysis of data from the other sequenced diatom, *Thalassiosira pseudonana*, helped identify several unique diatom genes that are specifically regulated under particular conditions, thus facilitating studies of gene function, genome annotation and the molecular basis of species diversity.

**Conclusions:**

The digital gene expression database represents a new resource for identifying candidate diatom-specific genes involved in processes of major ecological relevance.

## Background

In the current catalogue of eight major groups of eukaryotic taxa [1], the majority of well explored model organisms belong to the plant (Archaeplastida) and the animal (Opisthokonta) groups, which both evolved from primary endosymbiotic events that generated chloroplasts and mitochondria. The heterokonts, on the other hand, probably evolved from serial secondary endosymbiosis events in which a heterotrophic eukaryote engulfed both autotrophic red and green eukaryotic algae [2-4]. As a consequence, these organisms derive from the combination of three distinct nuclear genomes. The group includes highly diverse, ecologically important photosynthetic groups, such as diatoms, as well as non-photosynthetic members, such as oomycetes (for example, *Phytophthora infestans*, the causative agent of potato late blight).

Diatoms typically constitute a major component of phytoplankton in freshwater and marine environments. They are involved in various biogeochemical cycles, most notably those involving carbon, nitrogen and silicon, and contribute 30 to 40% of marine primary productivity [5,6]. Consequently, they are responsible for approximately one-fifth of the oxygen that is generated through photosynthesis on our planet. Morphologically, they exhibit different shapes and symmetries, the centric diatoms being radially symmetric and the pennates displaying bilateral symmetry. In spite of their tremendous ecological importance, the molecular mechanisms that enable them to succeed in a range of diverse environments remain largely unexplored.

Results from the first diatom genome projects from *Thalassiosira pseudonana *and *Phaeodactylum tricornutum *showed the presence of various genes needed for efficient management of carbon and nitrogen - for example, encoding urea cycle components [7,8]. However, these studies could only predict the functions of around 55% of diatom genes. The comparative study of the two diatom genomes [8] revealed that only 57% of genes are shared between the two diatoms, and that horizontal gene transfer from prokaryotes is pervasive in diatoms. Thus, the necessity for functional genomics and reverse genetics approaches to further explore diatom gene repertories is clear.

*P. tricornutum *is a pennate diatom that has been extensively studied physiologically and phylogenetically. In addition, it does not have an obligate requirement for silicic acid like other diatoms, and can undergo morphological transitions between three possible morphotypes [9]. The organism harbors a small genome (27.4 Mb) [8], it can be routinely transformed with efficiencies superior to those reported for other diatoms [10-13], and gene silencing is now possible using RNA interference [14]. For these reasons *P. tricornutum *is emerging as a model species for dissecting diatom molecular and cellular biology [15-20].

In a pilot study of the *P. tricornutum *genome using 1,000 cDNAs, only 23.7% of sequences could be functionally defined using homology-based methods [21]. This study was later expanded to 12,136 cDNAs [22], which facilitated comparative genomic studies of *P. tricornutum *with available genomes from the green alga *Chlamydomonas reinhardtii *[23], the red alga *Cyanidioschyzon merolae *[24], and the centric diatom *T. pseudonana *[7]. A number of interesting observations were made from such analyses about the evolutionary origins of individual genes [25]. This encouraged us to expand the EST repository by generating cDNA libraries from cells grown under different conditions of ecological relevance to increase the probability of obtaining unique gene expression profiles and to study the conditions in which they are induced. We describe herein statistical methods as well as comparative and functional studies to identify genes that are differentially expressed in 16 different conditions based on 132,547 cDNAs cloned and sequenced from *P. tricornutum*. These resources permit a systematic understanding of the molecular mechanisms underlying acclimation of this diatom to different nutrient conditions and its responses to various biotic and abiotic stresses, and should aid our understanding of the function of diatom-specific genes.

## Results

### Gene expression diversity across different cDNA libraries

To add to the previous 12,136 ESTs generated from cells grown in standard growth conditions (here denoted the 'OS library' for original standard [22], 15 non-normalized cDNA libraries were generated to explore the responses of *P. tricornutum *to a range of growth conditions, including different nutrient regimes of Si, N, Fe, and dissolved inorganic carbon (DIC), stress (hyposalinity and low temperature), and blue light. We also generated libraries from each of the three *P. tricornutum *morphotypes, and from cells exposed to the programmed cell death-inducing aldehyde decadienal [20]. The libraries were generated from three different ecotypes whose phylogenetic relationships and general characteristics have been previously described [26]. Furthermore, three different culturing regimes were used - batch, semi-continuous, and chemostats - depending on the treatment being performed. A comprehensive description of culturing conditions is provided in Materials and methods and Additional file 1, and is summarized in Table 1. To facilitate comparisons, all cells were harvested in mid-late exponential phase, and the libraries were made using the same RNA extraction and cDNA library construction methodologies (see Materials and methods).

**Table 1:** List of different libraries and culture conditions together with library statistics

Library	Short name	Strains	Condition/medium^a^	cDNAs	Contigs	Singletons	TUs	%R^b^
Original standard^c^	OS	Pt1 clone 8.6 (CCAP1055/1)		12,136	3,274	1,165	4,439	67.31
Silica plus	SP	Pt1 clone 8.6 (CCAP1055/1)	350 uM metasilicate in ASW	7,508	3,077	384	3,461	57.21
Silica minus	SM	Pt1 clone 8.6 (CCAP1055/1)	No metasilicate addition	6,968	2,838	459	3,297	54.63
Oval morphotype	OM	Pt3 (CCAP1052/1B)	Low salinity (10% ASW)	4,544	2,202	214	2,416	48.78
Nitrate replete	NR	Pt1 clone 8.6 (CCAP1055/1)	1.12 mM in chemostat	3,632	2,028	242	2,270	39.01
Nitrate starved	NS	Pt1 clone 8.6 (CCAP1055/1)	50 μM for 3 days in chemostat	9,122	3,271	512	3,783	60.79
Ammonium adapted	AA	Pt1 clone 8.6 (CCAP1055/1)	75 μM	9,031	3,329	567	3,896	60.20
Urea adapted	UA	Pt1 clone 8.6 (CCAP1055/1)	50 μM	8,552	3,157	464	3,621	59.82
Tropical accession	TA	Pt9 (CCMP633)	Grown at 15°C	4,821	2,015	160	2,175	56.95
Low decadienal	LD	Pt1clone 8.6 (CCAP1055/1)	0.5 μg/m 2E,4E-decadienal for 6 h	9,227	3,322	537	3,859	61.65
High decadienal	HD	Pt1 clone 8.6 (CCAP1055/1)	5 μg/m 2E,4E-decadienal for 6 h	3,541	1,734	323	2,057	44.95
Iron limited	FL	Pt1 clone 8.6 (CCAP1055/1)	5 nM	8,264	3,064	487	3,551	59.19
Triradiate morphotype	TM	Pt8 (CCAP1055)		12,566	3,055	520	3,575	70.49
Blue light	BL	Pt1 clone 8.6 (CCAP1055/1)	48 h dark adapted cells exposed to 1 h blue light	12,045	4,253	607	4,860	59.61
CO_2 _high 4 days	C4	Pt1 clone 8.6 (CCAP1055/1)	3.2 mM DIC for 4 days in chemostat	10,283	3,564	160	3,724	63.78
CO_2 _high 1 day	C1	Pt1 clone 8.6 (CCAP1055/1)	3.2 mM DIC for 1 day in chemostat	10,307	3,598	165	3,763	63.49

^a^All cells grown in artificial seawater media, except chemostat cultures, which were grown in Walne medium [54]. ^b^Percent redundancy of sequences in each library. ^c^The original *P. tricornutum *cDNA library described previously [21,22] is herein referred to as OS. Although incorporated into the comparative expression analyses, it was not examined extensively because it was generated using a different cDNA library protocol. ASW (artificial sea water),; TU, transcriptional unit.

**Figure 1:**
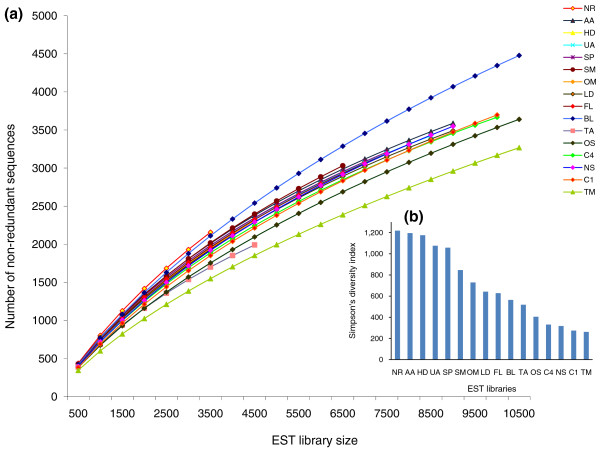
**Transcript diversity across libraries**. **(a) **Rarefaction curves of cDNAs sequenced from 16 different cDNA libraries. **(b) **Plot showing the Simpson's diversity index across the 16 libraries. For two-letter library codes, see Table 1.

The number of sequenced cDNAs per library varied from 3,541 to 12,566, with an average of 8,284 cDNAs per library for a total of 120,411 sequences. In general, the percentage of redundant sequences in the different libraries was around 50 to 60% (Table 1), although the triradiate morphotype (TM) library presented the highest level of redundancy (70%), whereas the lowest redundancy (39%) was observed in the nitrate replete (NR) library. Because the library size varied, we calculated rarefaction curves to check whether we had exceeded the optimal library size (that is, over sampling), which might have led to the redundancy variation [27]. All libraries were below saturation (Figure 1a), implying that further increases in library size would lead to the capture of new cDNAs. The differences in redundancy are not therefore due to over sequencing of some libraries. Consequently, the differences seen in the rarefaction curves along with the differences in redundancy rates of different libraries are likely to reflect differential gene expression in response to each culture condition.

To determine whether the abundance of transcripts was evenly distributed, that is, to check if the libraries have fewer sets of more abundant cDNAs (lower diversity) or several sets of evenly abundant cDNAs (higher diversity), we calculated the Simpson's reciprocal diversity index [28], which takes into account both the richness and evenness of transcripts in the libraries (the higher the index the higher the library diversity). Across the libraries we found the diversity index to vary from 1,218 to 268 (Figure 1b), with the nitrate replete (NR), ammonium adapted (AA), urea adapted (UA) and high decadienal (HD) libraries showing the highest diversity, and the nitrate starved (NS) and high CO_2 _(C1, C4) libraries showing the least diversity along with the most redundant triradiate morphotype (TM) library.

### Clustering of libraries and genes based on expression

We obtained a set of non-redundant transcriptional units (TUs) by aligning the 132,547 cDNAs with the 10,402 *P. tricornutum *predicted gene models using the BLAST program. A total of 11,513 sequences lacked predicted gene models and were clustered instead using CAP3 [29]. These represented a further 1,968 TUs in addition to the 8,944 TUs that aligned to the gene models [8]. In total, we obtained 9,145 transcripts present more than once across different libraries and 3,225 single copy transcripts, thereby comprising 12,370 TUs.

The top 20 most abundant transcripts are represented by cDNAs varying from 2,079 to 316 copies in all the 16 libraries (Table 2). The most abundant transcript (G49202), with 2,079 copies, belongs to a *P. tricornutum*-specific gene family (family ID 4628) with 9 members [8]. All nine encoded proteins contain predicted signal peptides and transcripts for them were detected in one or more cDNA libraries. They do not show any homology with known proteins (e-value cutoff = 10^-5^) with the exception of G49297, which shows some similarity to a bacterial protein containing a carbohydrate binding domain. When the above nine transcripts were subjected to PSI-Blast, we found a few transcripts showing low homology (e-value cutoff = 10^-3^, iterations = 3) to murine-like glycoprotein most typically associated with animal viruses. Eight of the genes belonging to the above gene family are localized on chromosome 21. The absence of this gene family in *T. pseudonana *and its high level of expression across various cDNA libraries may indicate that it represents a *P. tricornutum*-specific expanded glycoprotein gene family.

**Table 2:** Top 20 most highly expressed cDNAs across all the libraries, and their presence in different genomes

Contig	Cluster size^a^	G^b^	BLASTX description	InterPro description
G49202	2,079	P	-	-
G55010	856	P	-	Pyridoxal phosphate-dependent decarboxylase
G47667	833	O	Solute carrier family 34	Na+/Pi cotransporter
G27877	658	O	Ammonium transporter	Rh-like protein/ammonium transporter
G13951	630	C	Glutamate dehydrogenase	Glutamate dehydrogenase
G51797	613	D	Alpha 3 frustulin	-
G52619	605	O	Uric acid-xanthine permease	Xanthine/uracil/vitamin C permease
G44694	586	D	M6 family	Aldehyde dehydrogenase
G20424	561	O	Urea active isoform	Na+/solute symporter
G48027	545	P	-	-
G48315	479	V	Choline carnitine betaine transporter	BCCT transporter
G176.1	463	O	Alternative oxidase	Alternative oxidase
G29456	379	C	Glyceraldehyde-3- phosphate dehydrogenase	Glyceraldehyde 3-phosphate dehydrogenase
G49064	358	H	-	Na+/H+ antiporter NhaC
G49151	353	D	Nucleoside diphosphate epimerase	NmrA-like
G49211	346	P	-	-
C358	344	V	Periplasmic l-amino acid catalytic subunit	-
G30648	342	V	Light harvesting protein	Chlorophyll A-B binding protein
G23629	333	C	Calcium transporting ATPase	E1-E2 ATPase-associated region
G45835	316	V	-	Sterol-sensing 5TM box

^a^Cluster size of contig. ^b^Conserved in different representatives from eukaryotic genomes (e-value cutoff 10^-5^; more than 30% identity and 50% coverage): C, core (plant/animal/diatom); D, diatom (*P. tricornutum *and *T. pseudonana*); O, animal (opisthokonts); P, *Phaeodactylum tricornutum*; V, plant (Viridiplantae).

By comparing all of these highly expressed transcripts with those in 14 other eukaryotic genomes (see Materials and methods), we found that many are either present only in the two available diatom genomes or only in *P. tricornutum *(Table 2). Expression studies therefore represent a valuable resource for gene annotation in diatom and related genomes. Within the top 20 most abundant transcripts, some also encode highly conserved proteins such as glutamate dehydrogenase and glyceraldehyde-3-phosphate dehydrogenase, as well as others found in higher plants but not in animals (for example, ammonium transporter, light harvesting protein and alternative oxidase) (Table 2).

**Figure 2:**
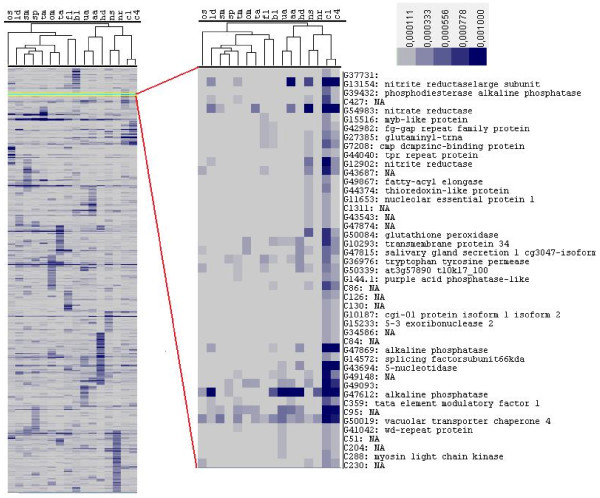
**Hierarchical clustering showing the expression pattern of transcripts expressed more than once in any of the 16 different growth conditions**. The blowup shows some of the genes differentially expressed in the high CO_2 _libraries (C1 and C4). Expression levels are shown in an increasing scale from grey to dark blue, and are based on frequencies of ESTs in each library (see Materials and methods). NA, no annotation information available. For two-letter library codes, see Table 1.

A range of different clustering and functional annotation methods was used to identify the libraries with similar gene expression patterns and to assess functional significance. We first made a hierarchical clustering [30] of the 9,145 transcripts expressed more than once, after normalizing transcript abundance in each individual library to library size. By this method we were able to identify libraries that share similar patterns of expression with reference to the presence or absence of a transcript and its relative abundance. Figure 2 shows the results visualized using 'Java Treeview' [31]. For example, from this analysis we see that libraries made from cells grown in chemostat cultures cluster together (NS, NR, C1 and C4). The oval morphotype (OM) and tropical accession (TA) libraries, which were derived from oval morphotypes grown at low salinity and low temperature, respectively, were also seen to cluster together.

We classified transcripts into three categories: core transcripts (represented across all 16 eukaryotic genomes), diatom-specific transcripts (expanded in the two available diatom genomes), and *P. tricornutum*-specific transcripts. Overall expression patterns of each class are similar (Additional file 2A), supporting the hypothesis that the diatom-specific genes do indeed represent *bona fide *genes. Furthermore, when expression patterns in individual libraries were explored, expression of these three classes of genes was seen to vary greatly (Additional file 2B). As an example, the aldehyde treated libraries (LD, HD) share a common pattern of expressed transcripts representing diatom-specific gene families (Additional files 2A and 3). A recurrent signature within this class of transcripts are stress-related protein domains associated with cell wall and membrane components, as well as proteases, lipases, glucanase, and elicitin. Expression analysis can therefore be used as a basis to explore the function of diatom-specific genes by comparing expression of the two diatom-specific classes of genes with the expression patterns of core genes. This comparison also demonstrates that the expression of core genes is generally higher when compared to the *P. tricornutum*-specific genes.

**Figure 3:**
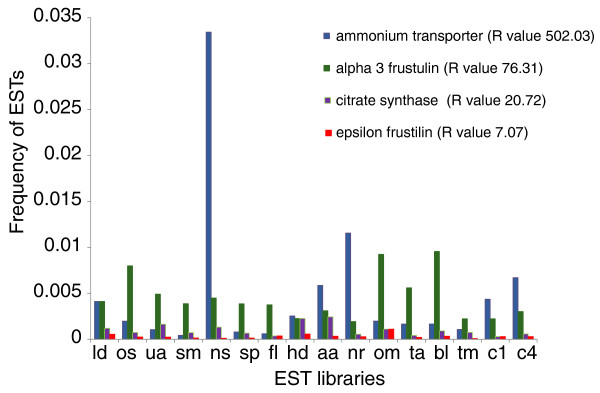
**Individual examples of expression patterns of transcripts that are expressed in all 16 conditions but with different log-likelihood ratios (R-values)**. For two-letter library codes, see Table 1.

While hierarchical clustering reveals the correlations and differences in patterns of gene expression across libraries, to identify transcripts that are differentially expressed, we used a statistical method based on log-likelihood [32]. For each TU we computed the log-likelihood ratio (R) and compared it with a randomly generated set (Additional file 4). Based on this comparison we considered TUs with R-values greater than 12 to be differentially expressed (see Materials and methods). On average, we detected between 200 and 450 differentially expressed transcripts per library (8 to 12%), the variation of which was mostly due to differences in library size (Additional file 5). Figure 3 shows examples of transcripts that are expressed across all 16 conditions and that have different R-values. An ammonium transporter encoding gene with an R-value of 502 was catalogued as being differentially expressed in the nitrate starved (NS) library, an alpha-3-frustulin encoding gene was catalogued as differentially expressed in the oval morphotype (OM) and blue light (BL) libraries, and a citrate synthase encoding gene was upregulated in the high decadienal (HD) and ammonium adapted (AA) libraries. By contrast, a gene encoding an epsilon-frustulin was not catalogued as being differentially expressed (R-value below 12). Seventy-one transcripts were expressed at least once across all the libraries (Additional file 6) and most of them were classified as being differentially expressed. Fifty-two of them also contained a known domain, and the majority fell into our category of core transcripts (30 sequences, against 15 diatom-specific transcripts, and 13 *P. tricornutum*-specific transcipts). These genes encode putative transporters (for bicarbonate and ammonium), some transcription factors, transposable elements, and the mitochondrial alternative oxidase, which has been proposed to be a central actor in diatom metabolism [33].

Based on our R-value criteria, only 7 genes could be defined as being constitutively expressed across all 16 libraries and these included frustulins and genes involved in cell division. This set of transcripts represents a valuable resource for promoter analysis, especially to identify constitutive promoters for reverse genetics studies.

### Gene Ontology term enrichment analysis

To further explore the functional significance of the library clusters and the differentially expressed genes in each library, functional annotation was performed using sequence and domain conservation analysis. For the transcripts showing sequence level similarity to 'known' proteins (Blastp, e-value <10 ^-5^), Gene Ontology (GO) term enrichment analysis was performed using blast2GO [34]. The GO terms of all the expressed transcripts were compared to the genes that are differentially expressed in each library. Additional file 7 shows the list of GO terms that are over-represented in each library (*P *< 0.001). In Additional file 7 we also show over-represented GO terms shared between libraries. The urea adapted (UA) and ammonium adapted (AA) libraries show over-representation of genes involved in nitrogen, amino acid, nucleotide and organic acid metabolism (Additional file 7), which is consistent with our knowledge of nitrogen metabolism. The blue light (BL) library contains the highest number of over-represented GO terms, and shares several categories related to photosynthesis and pigment biosynthesis with the iron limited (FL) library, such as porphyrin and tetrapyrrole biosynthesis. The significance of these shared terms with respect to metabolic management in iron starved cells has been discussed previously [33]. Additionally, the blue light library also has some unique GO terms, related to sugar and isoprenoid metabolism, transcription and translation, that likely reflect a general activation of metabolism stimulated by light exposure of dark-adapted cultures. These terms are not shared with other libraries.

The high decadienal (HD) library displays GO terms related to steroid metabolism as well as uncharacterized proteins involved in responses to biotic stimuli. These transcripts might provide insight into mechanisms of programmed cell death in diatoms because decadienal has been implicated in regulating the process [20,35]. The nitrate libraries (NR, NS) share a group of transporters and the nitrate replete (NR) library shows over-representation of nucleoside phosphate metabolic processes, specifically purine nucleoside triphosphate metabolism. The oval morphotype (OM) library, which is a salt stress library, shows over-representation of lipid metabolism classes whereas the triradiate morphotype (TM) library is over-represented in genes encoding active transport processes. In the high CO_2 _after 1 day (C1) library, COPI-vesicle-coat-related GO terms are over-represented, and in the high CO_2 _after 4 days (C4) library, inorganic anion transporters are over-represented. Perhaps surprisingly, in spite of clustering together in the hierarchical clustering analysis (Figure 2), the two high CO_2 _libraries (C1, C4) do not share any particular pathway terms. The over-representation of novel genes may be the reason for not finding any known GO terms between these two libraries, which illustrates our present ignorance of diatom biology, in spite of studying responses to a stimulus of significant ecological relevance.

### InterPro domain analysis

As an additional approach to examine the functional significance of differentially expressed transcripts, we explored domain content using InterPro [36]. We first classified putative proteins into two groups, those containing InterPro domains were denoted 'proteins with defined functions' (PDFs), and those with no recognizable domains were denoted 'proteins with obscure functions' (POFs) [37,38]. Comparisons with other organisms showed that most PDFs have orthologs in other heterokonts, particularly *T. pseudonana*, and that a significant number are also found in Viridiplantae and Opisthokonta (Additional file 8). Notwithstanding, a significant number of PDFs (1,011 out of 3,693) were not found in these 14 organismal groups compared, indicative of the highly chimeric nature of diatom genomes.

In a previous whole genome study of 10 different model eukaryotes, it was shown that POFs represent between 18 and 38% of a typical eukaryotic proteome [37]. In the putative proteome of *P. tricornutum *we found 44% of POFs, considerably more than usual, which likely reflects the fast evolving diatom genomes and the largely unexplored nature of diatom gene repertoires [8]. Table 3 shows the average protein composition statistics of the POFs and PDFs present in the *P. tricornutum *genome. We do not see higher variation in the length, amino acid composition and percentage of putative proteins with trans-membrane domains, indicating that the higher percentage of POFs is not likely to reflect pseudo-genes or transcripts that are not translated. We therefore propose that the majority encode *bona fide *genes.

**Table 3:** Average properties of encoded POF and PDF proteins in *P. tricornutum*

Protein property	POF	PDF
Length	440.6	477.4
Residue weight	110.9	110.8
Charge	11.4	10.8
Isoelectric point	7	6.9
Molecular weight	48,852.8	52,840.5
Transmembrane domains	1.424	1.487

Most of the differentially expressed transcripts encode PDFs; in particular, the blue light (BL) library contained more than 75% of proteins with defined domains, consistent with the fact that the BL library has the highest number of over represented GO terms (Additional file 5). This is possibly because we can infer a lot more about photosynthesis in diatoms by extrapolation of knowledge from plants and other algae than we can about other processes such as diatom responses to nutrients, which may therefore be rather novel. As a case in point, the most highly represented IPR domains in the blue light (BL) library included domains for bicarbonate transport, carbon fixation, light harvesting, and photosynthetic electron transport (Additional file 9), all of which are known to be key processes of photosynthesis.

**Figure 4:**
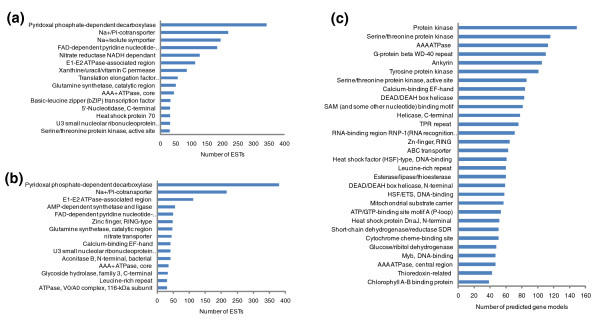
**InterPro domain representation of transcripts expressed in the high CO_2 _conditions**. **(a) **High CO_2 _after 1 day (C1); **(b) **high CO_2 _after 4 days (C4). **(c) **The 30 most highly represented InterPro domains across all the predicted gene models in the *P. tricornutum *genome shown for comparison.

As an example of using domain analysis to obtain functional information, the top 15 InterPro domains found in the high CO_2 _libraries (C1 and C4) are shown in Figures 4a,b. As a reference, Figure 4c shows the 30 most highly represented domains in the *P. tricornutum *genome, corresponding to gene families expanded in diatoms, such as protein kinases and heat shock transcription factors [8]. In the CO_2 _libraries we detected domains involved in pH maintenance and nitrogen metabolism, as well as a decarboxylase domain, found in just one gene. The function of this gene in diatom responses to high CO_2 _will be well worth exploring. The enlarged region in Figure 2 shows some of the other transcripts that are shared in the high CO_2 _conditions, including genes encoding nitrogen metabolism components. Genes involved in phosphate metabolism are also evident, suggesting that *P. tricornutum *responds to higher CO_2 _levels by up-regulating primary metabolic pathways.

The top 20 IPR domains in each of the other libraries are shown in Additional file 9. The data both confirm the validity of the culture conditions used for library generation (for example, the nitrogen libraries are over-represented in IPR domains related to nitrogen metabolism) and provide a new resource for exploring unanticipated aspects of diatom responses to specific stimuli. For example, the observed over-representation of IPR domains from heat shock transcription factors in these same libraries infers the importance of this class of transcription factors in regulating nitrogen metabolism.

### Correlations between libraries

**Figure 5:**
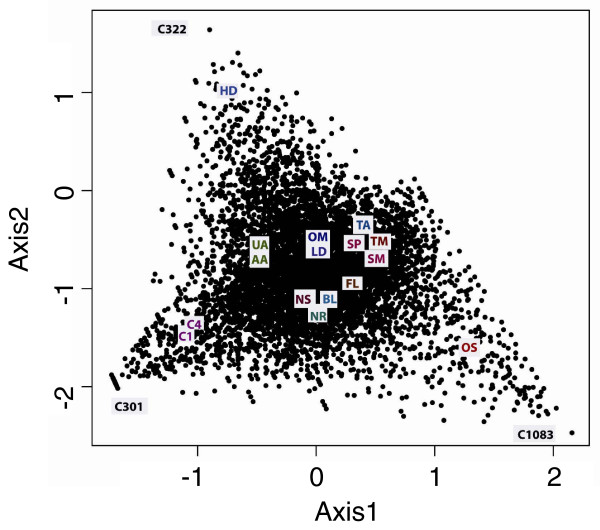
**Principle component analysis of all the libraries based on frequencies of expression across all 16 different conditions**. The plot shows the axes with maximum variation. For two-letter library codes, see Table 1.

Correspondence analysis (CA) was conducted with the 9,145 transcripts to identify correlated growth conditions. In this method, the frequencies of possibly correlated expression patterns are split into smaller components of un-correlated variables, and these components can be represented in multidimensional space using an axis for each transformed component. The first two components (axis) showing the maximum variance (least correlated) in expression are plotted in Figure 5. We found that the high decadienal (HD), original standard (OS) and high CO_2 _(C1, C4) libraries showed the maximum variance from the rest of the libraries. The dissimilarity of the OS library was not unexpected because it was created using different protocols compared to the other 15 libraries. It was therefore not considered further in this analysis. Comparative and functional analysis of the 100 genes showing maximum variance in expression in the other three conditions indicated that these transcripts mainly represent novel transcripts expressed in specific conditions and not predicted by conventional gene prediction programs or by other homology-based methods (data not shown). An example is shown in Figure 5, in which transcript C322 is unique to the high decadienal (HD) library and resembles a diatom-specific retrotransposon [39]. Conversely, transcript C301 is highly expressed uniquely in high CO_2 _conditions (C4 library), but does not have a predicted gene model. It does not show clear homology to any known sequence in other organisms, but its best BLAST hit is to a proteophoshphoglycan from *Leishmania *(data not shown). Interestingly, recent analyses have shown that this gene is also heavily methylated, unlike the majority of *P. tricornutum *genes (unpublished information Florian Maumus, Leila Tirichine and CB). Methylation of DNA is currently receiving attention as a mechanism controlling gene expression [40], so gene C301 is likely to be of great interest for future studies.

**Figure 6:**
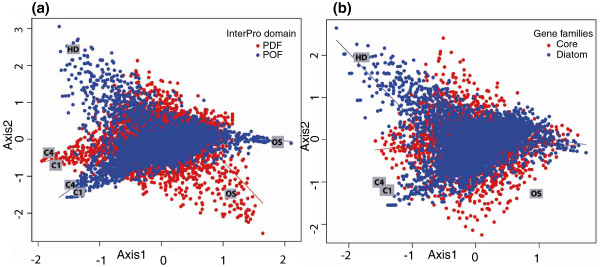
**Expression of POFs and of diatom-specific genes in all 16 libraries**. **(a) **Plot showing the axis 1 and axis 2 obtained from correspondence analysis of the expression of transcripts with known domains (PDFs) and without any predictable domain (POFs). **(b) **Plot showing the axis 1 and axis 2 obtained from correspondence analysis of the expression of transcripts that are conserved across 16 eukaryotic genomes (Core) and transcripts that are present only in diatom genomes (Diatom).

To further examine the contribution of known and unknown genes in each library, we repeated the correspondence analysis after classifying the transcripts based on the presence and absence of domains. Figure 6a shows that among the four libraries with maximum variation in expression, the high decadienal (HD) library displayed considerably more transcripts without a defined domain (POFs). The high CO_2 _(C1, C4) libraries have a roughly equal number of PDF and POF transcripts. Similar trends were seen when the analysis was repeated for diatom-specific transcripts (present in at least one of the two diatoms under study; e-value cutoff 10^-5^) or core transcripts also present in 14 other eukaryotic genomes (described in Materials and methods) (Figure 6b). We observed that the largest number of diatom-specific transcripts was found in the high decadienal (HD) library, followed by the high CO_2 _(C1, C4) libraries. These differences may imply that proteins with no recognizable homologs or domains may exhibit preferential involvement in species-specific regulatory and signaling networks [37]. As a case in point, the high decadienal treatment is known to induce programmed cell death and may be involved in regulating diatom population sizes [20,35].

### Expression analysis of diatom orthologous genes

**Figure 7:**
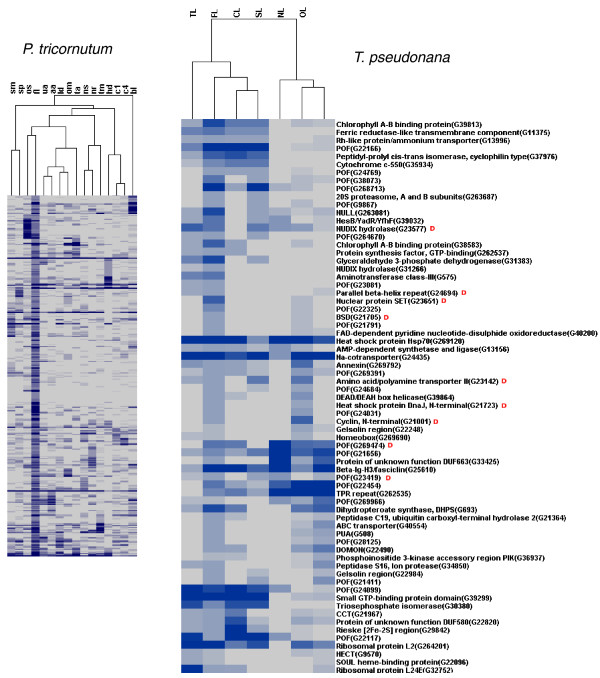
**Hierarchical clustering of transcripts defined as being differentially expressed under iron limitation (FL) in *P. tricornutum*, along with the hierarchical clustering of corresponding orthologs expressed under iron limitation (FL) in *T. pseudonana***. Expression levels are shown in an increasing scale from grey to dark blue, and are based on frequencies of ESTs in each library (see Materials and methods). For two-letter library codes for *P. tricornutum*, see Table 1. *T. pseudonana *library codes are TL, temperature limited; FL, iron limited; CL, carbon dioxide limited; SL, silicate limited; NL, nitrate limited; OL, old library; NP, nitrate plus [42]. The red letter 'D' in the *T. pseudonana *cluster denotes the diatom-specific transcripts.

The above described cDNA libraries from *P. tricornutum *are accessible through the diatom EST database, together with seven libraries from *T. pseudonana *[41]. Because two of the conditions were examined in both species (iron limitation (FL) and nitrogen starvation (NS) [42]), we could make a comparative analysis of the response of each diatom. A total of 346 and 278 transcripts were found to be differentially expressed in *P. tricornutum *under iron limitation (FL) and nitrogen starvation (NS) conditions, respectively. Among these transcripts, around 50% (174 in FL and 163 in NS) have orthologs in *T. pseudonana *(e-value cutoff 10^-5 ^) and a significant number of these are also responsive to the same treatment in this second diatom. Figure 7 shows hierarchical clustering of the 346 *P. tricornutum *FL transcripts together with 71 *T. pseudonana *putative orthologs that are also expressed under iron limitation (FL). Within this set we can find diatom-specific POFs as well as transcripts with recognizable domains such as transcription factors (Figure 7). We can also find genes encoding photosynthetic components and putative cell wall proteins (fasciclin, gelsolin, annexin), implying that the global reprogramming of cellular metabolism observed in *P. tricornutum *[33] may be common to other diatoms as well. In a similar analysis performed with the 163 *T. pseudonana *orthologs of the nitrate starvation responsive *P. tricornutum *genes, 46 were found to be differentially expressed in the same condition in *T. pseudonana *(Additional file 10). These include genes encoding components of nitrogen metabolism, regulatory pathways, and a range of POFs. Only one of the genes expressed in response to nitrate starvation in both diatoms is diatom-specific, whereas nine of the iron responsive genes were classified as being diatom-specific (compare Figure 7 and Additional file 10). This could suggest that diatom responses to iron have evolved specifically in diatoms, whereas nitrate starvation responses may constitute a more general organismal response.

Whole-genome expression profiling using a tiled array in *T. pseudonana *led to the identification of previously un-annotated TUs [42]. Among these 3,470 TUs, 1,458 were also found in the *P. tricornutum *genome (e-value cutoff 10^-5^), and of these, 1,086 were expressed under various conditions in *P. tricornutum*. Additional file 11 shows the expression patterns of these genes and it is apparent that many of these TUs are highly expressed in the high decadienal (HD) cDNA library. This result is consistent with the previous observations revealing the unique expression patterns of diatom-specific gene families and 'unknown' genes in the HD library (for example, Figure 6).

### Expression patterns of bacterial genes

It was previously reported that horizontal gene transfer from bacteria is one factor affecting diatom genome diversity, with at least 587 genes of proposed bacterial origin were identified in the *P. tricornutum *genome [8]. The expression of these genes was analyzed to study the functional significance of these acquired genes. A total of 446 bacterial genes were expressed under various growth conditions, and 50% of them were expressed in the blue light (BL) library (Additional file 12A). The most highly expressed bacterial genes encode a putative Na+/H+ antiporter, hybrid cluster protein, and nitrite reductase (Additional file 13). The latter two have been discussed previously in the context of their importance for nitrogen metabolism in diatoms [43]. In spite of having fewer numbers of expressed bacterial genes, higher frequencies of certain cDNAs were found in the oval morphotype (OM) and tropical accession (TA) libraries. The functional significance of these bacterial genes was explored with reference to their orthologs in other bacterial genomes using the COG database of bacterial orthologous gene clusters [44]. The set of bacterial genes identified in *P. tricornutum *were found to represent 19 different COG classes (Additional file 10), with genes belonging to 'energy production and conversion' being the most highly expressed. By contrast, genes belonging to the categories of intracellular trafficking, secretion, cell motility, and chromatin structure were under-expressed.

### Gene composition and expression

**Figure 8:**
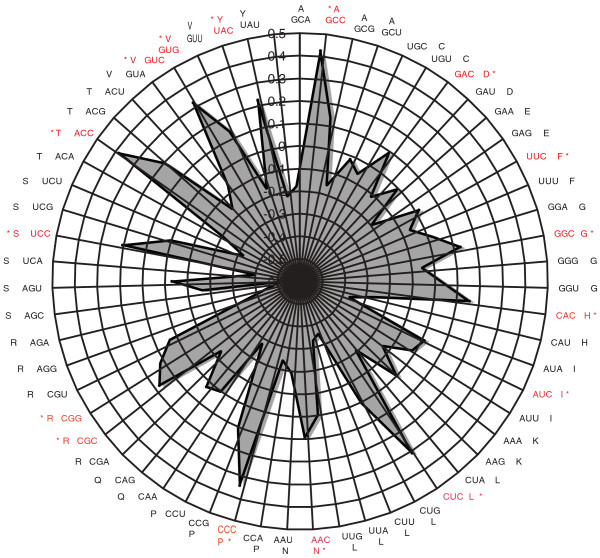
**Radar plot showing the preference of C ending codons, obtained from Codon Adaptation Index analysis done on highly expressed transcripts only**. Codons shown in red correspond to the most commonly used codons.

In a pilot analysis with the 12,136 cDNAs from the OS library, it was shown that transcripts that are represented by higher numbers of ESTs show higher levels of guanine and cytosine nucleotides at the third codon position (GC3) [25]. To examine the significance of correlation between expression level and codon usage bias, we derived a codon usage table using the predicted gene models for *P. tricornutum*, which is available at [45]. We applied correspondence analysis to study the relative synonymous codon usage [46] in the highly expressed genes across all the libraries. Codon Adaptation Index values were then calculated using CodonW [47]. Correspondence analysis allowed the identification of the first four axes (components) that explain the majority of the variance (30.6%) in codon usage among *P. tricornutum *putative coding sequences (CDSs). The principal axis (F1) contributes 15.5% to the total variance, while the second axis (F2) explains only 6%. Consequently, we can conclude that F1 is the main factor driving codon usage heterogeneity in *P. tricornutum*. We therefore chose the projection of points (the 59 codons) along F1 and used these coordinates as an estimator of the relative usage of degenerate codons within each CDS (Figure 8). The preferred codons are generally C-ending, in agreement with previous reports [25]. Therefore, C-ending codons are likely to be translationally optimal. An additional interesting feature of *P. tricornutum *CDSs is the relative lack of G3 ending codons among quartets.

## Discussion

The *P. tricornutum *cDNAs described in this report were obtained from cells grown in 16 different conditions of ecological relevance, and are publicly available in a digital gene expression database [48]. In total, they correspond to 86% of the predicted genes in the genome, and are therefore a useful basis for exploring gene expression patterns. As demonstrated here they can also be used to probe the function of genes that do not show significant homology to transcripts in other sequenced genomes. How many of the remaining 14% of *P. tricornutum *gene models that lack EST support actually represent *bona fide *genes is unknown [8]. The fact that we could detect an additional 1,968 TUs that lack gene models shows the limitations of current gene prediction programs to detect diatom genes, and sets an upper limit of 12,370 genes in *P. tricornutum*, in the same range as the upper count of 14,862 genes predicted by expression analysis in *T. pseudonana *[42]. The number of diatom genes that encode small RNAs rather than proteins is also unclear at this time, although the expressed *P. tricornutum *genes that lack homology to known sequences do appear in general to encode proteins with the typical biochemical characteristics of *P. tricornutum *proteins (Table 3).

Due to the phylogenetic distance of diatoms from most of the eukaryotes for which whole genome sequences are available, comprehensive cDNA collections also provide an important resource to improve gene prediction. For example, in *P. tricornutum *only 28% of the gene models could be predicted by homology-based methods; the others were predicted using the cDNAs reported here as a training set for *ab initio *methods [8]. This data set will also be of importance for the growing number of diatom genome projects, for example, from *Pseudo-nitzschia multiseries *and *Fragilariopsis cylindrus*, as well as for other heterokont sequencing projects.

An important aspect of the current study is that 15 of the libraries were generated from non-nomalized mRNA populations and using the same methodologies (the original library (OS) described previously in [21] was generated using a different method). The gene expression patterns in each culture condition can therefore be compared and contrasted with the other conditions. To facilitate this, we converted EST counts to frequencies in each library, examined redundancy by rarefaction, and diversity using Simpson's index. Although all libraries were clearly under-saturated, there was wide variation in redundancy and diversity (Figure 1). Some libraries were characterized by having several sets of evenly abundant cDNAs - for example, the nitrate replete (NR) library - while others had fewer sets of highly abundant cDNAs - for example, the nitrate starved (NS) library. These results therefore provide information about how *P. tricornutum *responds to the different conditions examined.

Although the 15 cDNA libraries were generated and sequenced using the same protocol, a potential caveat of our approach is that the culturing conditions under which the libraries were generated were not identical (Additional file 1) because they were generated in different laboratories worldwide. To reduce unnecessary heterogeneity, all cells were nonetheless harvested at mid- or late exponential phase. Furthermore, in our opinion the results from our statistical analyses demonstrate clearly the biological significance of the measured transciptional outputs - for example, the nitrogen limited libraries show clear effects in genes involved in nitrogen metabolism.

Our analyses are especially valuable for the exploration of diatom genes with undefined functions because expression profiling can shed light on their functional significance [49]. Many of these genes encode proteins that lack recognizable InterPro domains, and have been classified as encoding POFs. Diatom genomes encode higher numbers of POFs than have been observed in other genomes (see Results). In rice and *Arabidopsis*, such genes are thought to contribute to ecological differences and species diversity [37,38]. The predicted biochemical characteristics of these putative *P. tricornutum *proteins suggest that they represent functional proteins. Approximately half of them are also found in *T. pseudonana*, and interestingly can be seen in many cases to be specifically induced by high decadienal treatment (Figure 6; Additional file 11). This aldehyde is of interest because it has been implicated in regulating diatom population densities [20,50], so these genes deserve attention as being of potential importance in the control of population density and programmed cell death.

The different statistical methods employed in this study provide support for several recent hypotheses proposed on the basis of experimental observations; for example, the commonalities of nitrate, ammonium and urea assimilation [7,43] can be seen in the similar expression profiles of the NS, AA, and UA libraries, and the reprogramming of diatom photosynthesis in response to iron limitation [33] is reflected in the common gene expression profiles between the FL library and the blue light (BL) library (Figure 2). Conversely, the two abiotic stress libraries - low temperature (TA) and low salinity (OM) - display similar expression profiles (Figure 2), in agreement with the known overlap in the response to these stresses in other organisms [8]. These expected results are satisfying, but more importantly they increase confidence that the methodologies used can help resolve other less well understood processes involved in each individual response. For example, when hierarchical clustering is done using only the small set of 177 expressed transcription factors in *P. tricornutum*, the relationships observed between the different libraries are essentially the same as can be seen when using all 9,145 TUs [17] (Figure 2). Hence, the methodologies reported here can help identify transcription factors associated with differential expression in the different growth conditions. Conversely, genes of unknown function can be recruited to a specific response, for example, those induced in response to high decadienal. Such correlations provide a reasonable basis to explore the function of such genes.

Finally, our studies have helped to understand better the roles in diatoms of genes of probable bacterial origin. These acquired bacterial genes have undergone modifications, such as gene fusions and novel domain reorganizations [8]. The observed diversity of their phylogenetic origins, derived from a wide range of different bacterial groups, is reflected in their functional diversity in that they belong to 19 different COG categories. Many are expressed in different conditions (Additional file 12), which may reflect the functional adaptation of acquired bacterial genes to important metabolic and regulatory processes. In other cases, they are not strongly expressed - for example, genes in the intracellular trafficking, cell motility, secretion, and chromatin structure categories - which may indicate that genes derived from the secondary endosymbiotic parents have been retained for these functions.

Although there can be no substitute for laboratory-based exploration of gene function, the sheer numbers of diatom-specific genes of unknown function necessitates the availability of high throughput *in silico *approaches that can allow diatom researchers to identify interesting candidate genes that likely play key functions. We believe that the resources described here represent a significant step forward for characterizing diatom genes, as highlighted by PtTU G49202, a *P. tricornutum *gene of unknown function that is extremely highly expressed (Table 2), and PtTU G55010, a carboxylase of unknown function that we have linked to the diatom response to high CO_2_. Reverse genetics approaches such as RNA interference [14] can now be directed towards genes such as these in order to define their precise functions experimentally and to help understand the specific innovations that have led to the dominance of diatoms in contemporary ecosystems.

## Conclusions

The unbiased statistical methods used in the current study to analyze diatom gene expression profiles in different conditions can provide insights of biological relevance for understanding how diatoms respond to their environment, and in particular can pinpoint genes with unassigned functions for targeted studies. The digital gene expression database that we have established [48] represents a new resource for identifying candidate diatom-specific genes involved in processes of major ecological relevance.

## Materials and methods

### Library conditions

A total of 15 cDNA libraries were constructed to explore the molecular responses of *P. tricornutum *to a range of conditions, in addition to the previously reported library [21]. The different libraries are summarized in Table 1 and Additional file 1, and the rationale for choosing each condition is summarized below. All libraries are derived from cells in mid-late exponential phase.

Geographically widely distributed strains of *P. tricornutum *show interesting intra-species genetic and phenotypic diversity [26] and undergo morphological transitions between three possible morphotypes [9]. To explore the functional adaptations of different morphotypes, we made libraries from each of them. The triradiate morphotype (TM) library was constructed from mRNAs extracted from cells grown by repeated subculturing of the Pt8 accession under gentle agitation (80 rpm) in order to obtain a culture with 70% of triradiate cells. The oval morphotype (OM) library was made using the Pt3 accession grown at low salinity, and the tropical accession (TA) library was obtained from Pt9 cells grown at low temperature (15°C), treatments that both induce the formation of oval cells [26]. Cells were obtained from cultures either following a shift to hyposalinity 10% in Brown's artificial sea water (ASW) [26] for Pt3 or after a shift from 28°C to 15°C for Pt9.

The Pt1 8.6 accession also produces oval morphotype cells when treated with decadienal, a programmed cell death-inducing aldehyde [35]. Chemical signaling induced by this diatom-derived aldehyde has been shown to trigger stress responses and may control bloom dynamics in phytoplankton populations [20]. Two libraries, low decadienal (LD) and high decadienal (HD), were constructed to explore the genes expressed in response to sublethal (0.5 μg/ml) and lethal (5 μg/ml) concentrations of decadienal after 6 h treatment, respectively.

All other libraries were derived from cultures of the Pt1 8.6 accession grown under different nutrient regimes that contained predominantly the fusiform morphotype. Iron bioavailability is a major factor limiting photosynthetic biomass in the ocean [51] and Fe fertilization experiments have shown that diatoms show greater sensitivity to iron compared to other phytoplankton [52]. The iron limited (FL) library was designed to study genes expressed at low concentrations of iron [33].

Diatoms also play a major role in nitrogen-based biogeochemical cycles, and the diatom genome has been shown to encode various nitrate and ammonium transporters as well as the full complement of urea cycle enzymes [7,43]. cDNA libraries were therefore constructed from cells grown under various nitrogen regimes to help understand the complexities of nitrogen metabolism in diatoms. We constructed four such libraries from nitrate-starved (NS) cells grown in 50 μM of nitrate for 3 days, nitrate-replete (NR) cells grown in 1.12 mM nitrate, and from ammonium adapted (AA) and urea adapted (UA) cultures. The NS and NR cultures were grown under continuous light (120 μmol.m^-2^.s^-1^). Walne medium [53] was used for the NR condition and NO_3_^- ^concentration was reduced to 50 μM for the NS condition. Cultures were sampled at both steady state (NR and NS) as well as during the N-depletion period.

Additionally, cDNAs obtained from cells grown in the presence and absence of silicic acid (SP and SM, respectively), formed a useful data set for studies of the silicification process [54].

To examine light-responsive gene expression, a cDNA library was generated from cultures grown in 12 h light-dark cycles to a concentration of 0.5 × 10^6 ^cells per milliliter, subsequently dark adapted for 60 hours, and then exposed to blue light. Cells were harvested after 1 h induction by centrifugation for 15 minutes at 3,000 *g *and conserved at -80°C until RNA extraction.

Diatoms are responsible for about 20% of global carbon fixation and many studies are underway to explore how diatoms may be affected by climate change. We therefore constructed cDNA libraries from cells grown under high carbon dioxide concentrations for one (C1) and 4 days (C4). The study of adaptation to such conditions is of particular interest in the light of increasing CO_2 _levels in the Earth's atmosphere. For these experiments cells were initially grown at 2.8 mM DIC with a dilution rate of 0.5 d^-1 ^(temperature and pH were kept constant at 20°C and 8.0, respectively), and high CO_2 _conditions were obtained by increasing DIC to 3.2 mM by bubbling carbon dioxide into the cultures until the pH reached 7.0.

Moreover, we used three different culture methods; the NS, NR, C1 and C4 libraries were all grown in chemostat cultures, whereas the OM, AA, UA, TA, FL, and other libraries were from batch cultures. For the carbon dioxide chemostat experiments the cells were grown at 20°C under continuous light (120 μmol.m^-2^.s^-1^) with an operating speed of 100 rpm. The cultures were run at 20% dilution rates and sampled at steady state at pH 8.0 and pH 7.0 as well as during the adjusting period between these two levels, that is, within 24 h after pH modification.

The above libraries can therefore be used to study growth conditions and gene expression in response to different stimuli of ecological relevance. Although the cDNA library that we originally characterized [21] was also incorporated into the current analyses, certain comparisons with the other libraries should be viewed with caution because this cDNA library was constructed using different methodologies.

### Library construction

The non-normalized cDNA libraries were constructed from poly(A)^+ ^RNA purified from total extracted diatom RNA using the CloneMiner cDNA library construction kit (Invitrogen, Cergy-Pontoise, France) following the supplier's instructions with minor modifications. Fifteen different conditions (Table 1) were used to maximize the detection of genes expressed with specific condition-enriched profiles. Sequencing was performed mostly from the 5′ end of the insert but for some of the libraries an attempt was made to sequence each clone at both the 5′ and the 3′ ends. When both EST reads overlapped, the two sequences were fused into a consensus sequence using PHRAP [55].

### Sequence analysis

The complete set of cDNAs was subject to a preliminary analysis as previously described in [22], with a variation in obtaining the non-redundant data set. The cDNAs were first aligned to the predicted gene models available at the Joint Genome Institute (JGI) [56] using the BLAST program [57] and the cDNAs that did not have a predicted gene model were subjected to CAP3 assembly [29]. This two step procedure to derive the non-redundant set avoided the over-estimation of non-redundant transcripts (TUs) led by short transcripts. The cluster size (that is, the number of redundant cDNAs for each TU) was obtained and the number of transcripts contributed by each individual library to cluster size was also counted for all the TUs. An initial functional annotation of the non-redundant transcripts was done using blast2GO [34]. A more advanced annotation, such as the assignment of InterPro domains and KEGG pathways, was obtained from the *P. tricornutum *genome annotation performed at the JGI.

The *P. tricornutum *and *T. pseudonana *sequences were also compared by BLASTX to those in 14 other eukaryotic genomes, specifically *Phytophthora ramorum*, *Phytophthora sojae*, *Chlamydomonas reinhardtii*, *Ostreococcus lucimarinus*, *Ostreococcus tauri*, *Cyanidioschyzon merolae*, *Monosiga brevicollis*, *Dictyostelium discoideum*, *Ciona intestinalis*, *Caenorhabditis elegans*, *Aspergillus niger*, *Pichia stipitis*, *Arabidopsis thaliana *and *Saccharomyces cerevisiae*.

### Library richness and diversity

The richness and diversity of cDNAs sampled from each cDNA library was estimated by statistical methods. Richness was estimated by rarefaction using the Analytic Rarefaction 1.3 program [58] to plot the rarefaction curve. Diversity was estimated using Simpson's Reciprocal Index and was calculated using the formula (1/D), where D is Simpson's index calculated using the formula [28]:

$$\text{D}=\frac{\sum \text{n}(\text{n}-1)}{\text{N}(\text{N}-1)}$$

where n is the cluster size (the number of cDNAs of each TU in each library) and N is the library size (the total number of cDNAs sequenced in each library).

### Data normalization and clustering

The count of cDNAs in each library and for each cluster (TU) was normalized to the library size by calculating the frequency (the EST count divided by the library size). This normalized data facilitated the comparisons of expression across different libraries in spite of the differences in library sizes. The frequency distribution of 9,145 TUs that are expressed more than once across one or more libraries were used for principal component analysis. Principal component analysis was done using R version 2.5.0 [59]. The same data set was used to identify genes and libraries with similar expression patterns using two-way hierarchical clustering. The hierarchical clustering was done using the program cluster 2.11 [30] and was visualized using 'Java Treeview' [31].

### Differential gene expression

To study the distribution of transcripts across different libraries and to identify clusters that were significantly over-represented in certain growth conditions, we examined the expression patterns of the 9,145 clusters. To eliminate differences in distribution caused by the differences in library size, the counts were normalized by converting them to frequency. To determine whether the differences in frequency distribution were due to statistically significant differential gene expression or to a random distribution, we calculated the log likelihood ratio, R-value [32], for each cluster. To define a cutoff R-value, we calculated the R-value for 9,145 random clusters generated from a Poisson distribution whose parameter is equal to the expected cluster size for that library 1,000 times. Additional file 4 shows the R-values of the actual 9,145 clusters and in the randomized data set. The probability of having an R-value above 23 was zero in all randomized clusters. We considered an R-value cutoff of 12, corresponding to 0.96 probability (that is, with 96% chance that it is not a random event), providing a useful basis to define genes displaying differential mRNA levels in our cDNA libraries. The 8,402 transcripts with R-values above 12 were analyzed and catalogued as differentially expressed in the libraries having the top two highest frequencies. Transcripts represented by three or fewer cDNAs were removed.

### Gene Ontology and COG annotation

The expressed TUs were subjected to GO annotation [60] and GO term enrichment analysis was done using the Blast2GO program [34]. COG annotation was done by RPS-BLAST [61] with the TUs as query using the Conserved Domain Database (CDD) [62]. COG identities were assigned to any TU with a BLAST e-value below 10^-3^.

### Codon usage analysis

The codon usage table for *P. tricornutum *was obtained using the CDSs from JGI and using the EMBOSS program [63]. Codon Adaptation Index values were calculated using CodonW [47]. This analysis was performed using all the CDSs starting with ATG, and the maximum number or the sum of cDNAs across all the libraries was taken as an expression level index. Genes represented by more than 30 cDNAs were used as a reference set for highly expressed genes to study codon usage bias and its relation to expression level.

## Abbreviations

AA: ammonium adapted; ASW: artifical sea water; BL: blue light; C1: high CO_2 _1 day; C4: high CO_2 _4 days; CDS: coding sequence; DIC: dissolved inorganic carbon; EST: expressed sequence tag; FL: iron limited; GO: Gene Ontology; HD: high decadienal; JGI: Joint Genome Institute; LD: low decadienal; NR: nitrate replete; NS: nitrate starved; OM: oval morphotype; OS: original standard; PDF: protein with defined function; POF: protein with obscure function; SM: silicate minus; SP: silicate plus; TA: tropical accession; TM: triradiate morphotype; TU: transcriptional unit; UA: urea adapted.

## Authors' contributions

UM performed the bioinformatics analyses and prepared the manuscript, KJ performed codon usage analysis and provided advice on data interpretation, JLP, BMP, JW, and MK perfected RNA extraction and library construction, and performed DNA sequencing, assembly and annotation, AEA, JPC, ADM, MH, RK, JLR, PJL, VMJ, AM, TM, MSP and AV prepared cultures and provided critical advice on analysis outputs, EVA helped in data interpretation, CB coordinated the work and finalized the manuscript. All authors have read and approved the manuscript.

## Supplementary Material

Additional file 1 **Supplementary Table S1**. A comprehensive description of culturing conditions of the libraries. Click here for file

Additional file 2 **Supplementary Figure S1**. Expression patterns of diatom-specific genes. **(A) **Hierarchical clustering to show the expression pattern of transcripts belonging to the gene families conserved across different taxonomical groups (Core), diatom-specific (Diatom) and *P. tricornutum-*specific (Pt) [8]. **(B) **Plot showing the average frequency of the above set of transcripts across the 16 different conditions. In (A), expression levels are shown in an increasing scale from grey to dark blue, and are based on frequencies of ESTs in each library (see Materials and methods). For two-letter library codes, see Table 1. Click here for file

Additional file 3 **Supplementary Table S2**. Diatom-specific genes expressed in both high and low decadienal libraries (HD and LD). Click here for file

Additional file 4 **Supplementary Table S3**. R-values of the actual 9,145 clusters and that of the randomized data set. Click here for file

Additional file 5 **Supplementary Figure S2**. Percentage of differentially expressed transcripts in primary y-axis, normalized to number of non-redundant transcripts (TUs) across the EST libraries and the percentage of transcripts with defined InterPro domains (PDFs) in the differentially expressed transcripts in the secondary y-axis. The arrow in the secondary y-axis at 56% corresponds to the percentage of PDFs found in all the putative proteins predicted in the *P. tricornutum *genome (5,825 out of 10,402 protein models). For two-letter library codes, see Table 1. Click here for file

Additional file 6 **Supplementary Table S4**. The 71 transcripts that were expressed at least once across all the libraries. Click here for file

Additional file 7 **Supplementary Table S5**. GO terms that are over-represented in each library (*P *< 0.001). In this table we also show over-represented GO terms shared between libraries. Click here for file

Additional file 8 **Supplementary Figure S3**. Distribution of *P. tricornutum *PDFs in other organismal groups. Numbers in parentheses indicate the number of genes with defined protein domains (PDF) and the number outside the parentheses represent the total number of genes in each organismal group. Click here for file

Additional file 9 **Supplementary Table S6**. The top 20 IPR domains expressed across all the libraries and the number of ESTs for each domain. Click here for file

Additional file 10 **Supplementary Figure S4**. Hierarchical clustering of transcripts defined as being differentially expressed under the nitrate starved condition (NS) in *P. tricornutum *along with the hierarchical clustering of corresponding orthologs expressed in the nitrate limited condition (NL) in *T. pseudonana*. Expression levels are shown in an increasing scale from grey to dark blue, and are based on frequencies of ESTs in each library (see Materials and methods). For two-letter library codes, see Table 1 and the Figure 7 legend. Click here for file

Additional file 11 **Supplementary Figure S5**. Hierarchical clustering showing the expression patterns of *P. tricornutum *orthologs of the novel genes identified by tiling array in *T. pseudonana *[42]. Expression levels are shown in an increasing scale from grey to dark blue, and are based on frequencies of ESTs in each library (see Materials and methods). For two-letter library codes, see Table 1. Click here for file

Additional file 12 **Supplementary Figure S6**. Expression of bacterial orthologous genes in *P. tricornutum*. (A) Plot showing the number of transcripts of bacterial origin expressed across the 16 different growth conditions. The primary y-axis shows the number of transcripts and the secondary y-axis shows the average frequency of these expressed transcripts. **(B) **Expression profiling of the genes of putative bacterial origin along with their COG categories. The gradient of blue shows the level of expression, with the darker colors being the highly expressed genes. The red color shows the lack of expressed transcripts. For two-letter library codes, see Table 1. Click here for file

Additional file 13 **Supplementary Table S7**. Bacterial genes and their expression across different libraries along with the domain and genomic location. Click here for file
