# Supplementary figures and images for: Digital expression profiling of novel diatom transcripts provides insight into their biological functions
Source: Genome Biol. 2010 Aug 25;11(8):R85. doi: 10.1186/gb-2010-11-8-r85 (PMC2945787; doi:10.1186/gb-2010-11-8-r85)

Figure S1

A

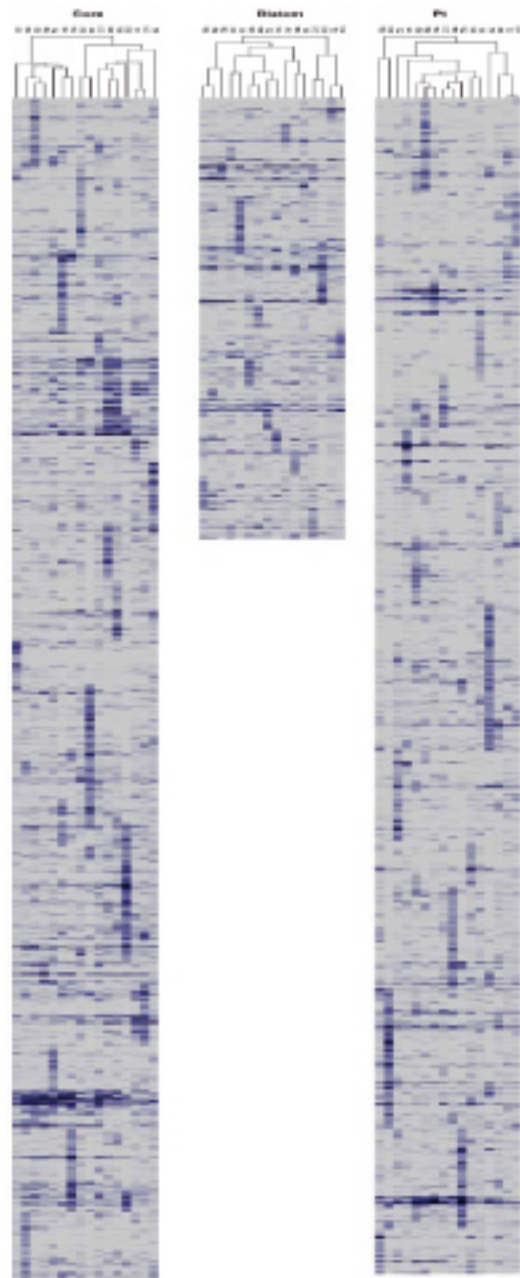

B

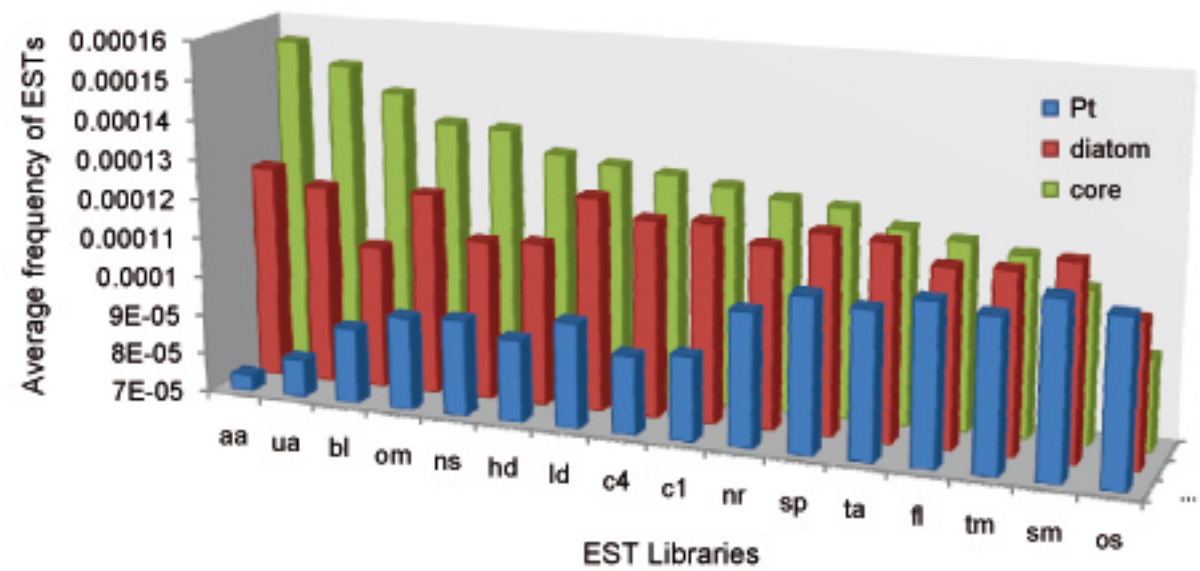

Supplement: Additional file 2 — Supplementary Figure S1. Expression patterns of diatom-specific genes. (A) Hierarchical clustering to show the expression pattern of transcripts belonging to the gene families conserved across different taxonomical groups (Core), diatom-specific (Diatom) and P. tricornutum-specific (Pt) [8]. (B) Plot showing the average frequency of the above set of transcripts across the 16 different conditions. In (A), expression levels are shown in an increasing scale from grey to dark blue, and are based on frequencies of ESTs in each library (see Materials and methods). For two-letter library codes, see Table 1. [file gb-2010-11-8-r85-S2.pdf]

Figure S2

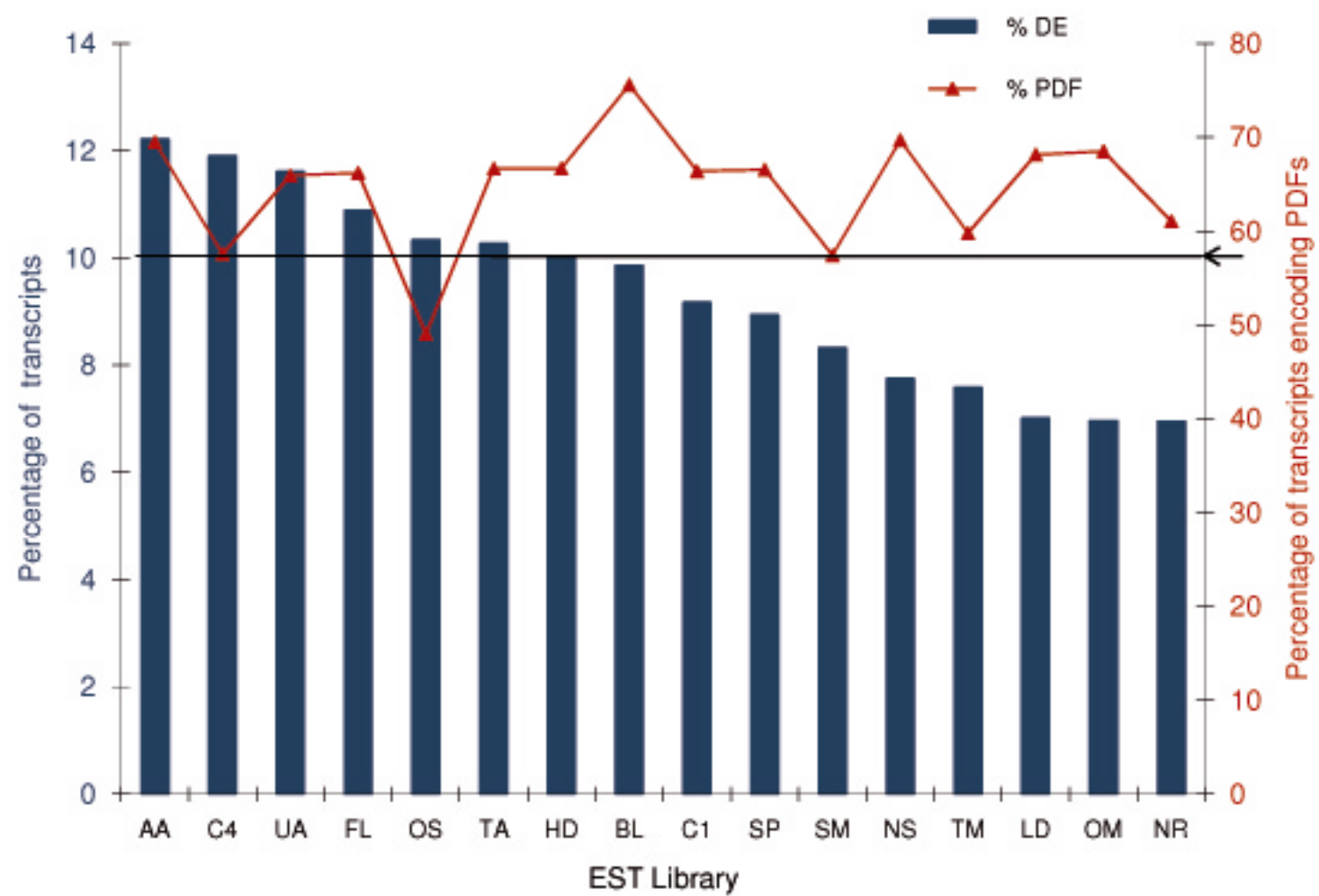

Supplement: Additional file 5 — Supplementary Figure S2. Percentage of differentially expressed transcripts in primary y-axis, normalized to number of non-redundant transcripts (TUs) across the EST libraries and the percentage of transcripts with defined InterPro domains (PDFs) in the differentially expressed transcripts in the secondary y-axis. The arrow in the secondary y-axis at 56% corresponds to the percentage of PDFs found in all the putative proteins predicted in the P. tricornutum genome (5,825 out of 10,402 protein models). For two-letter library codes, see Table 1. [file gb-2010-11-8-r85-S5.PDF]

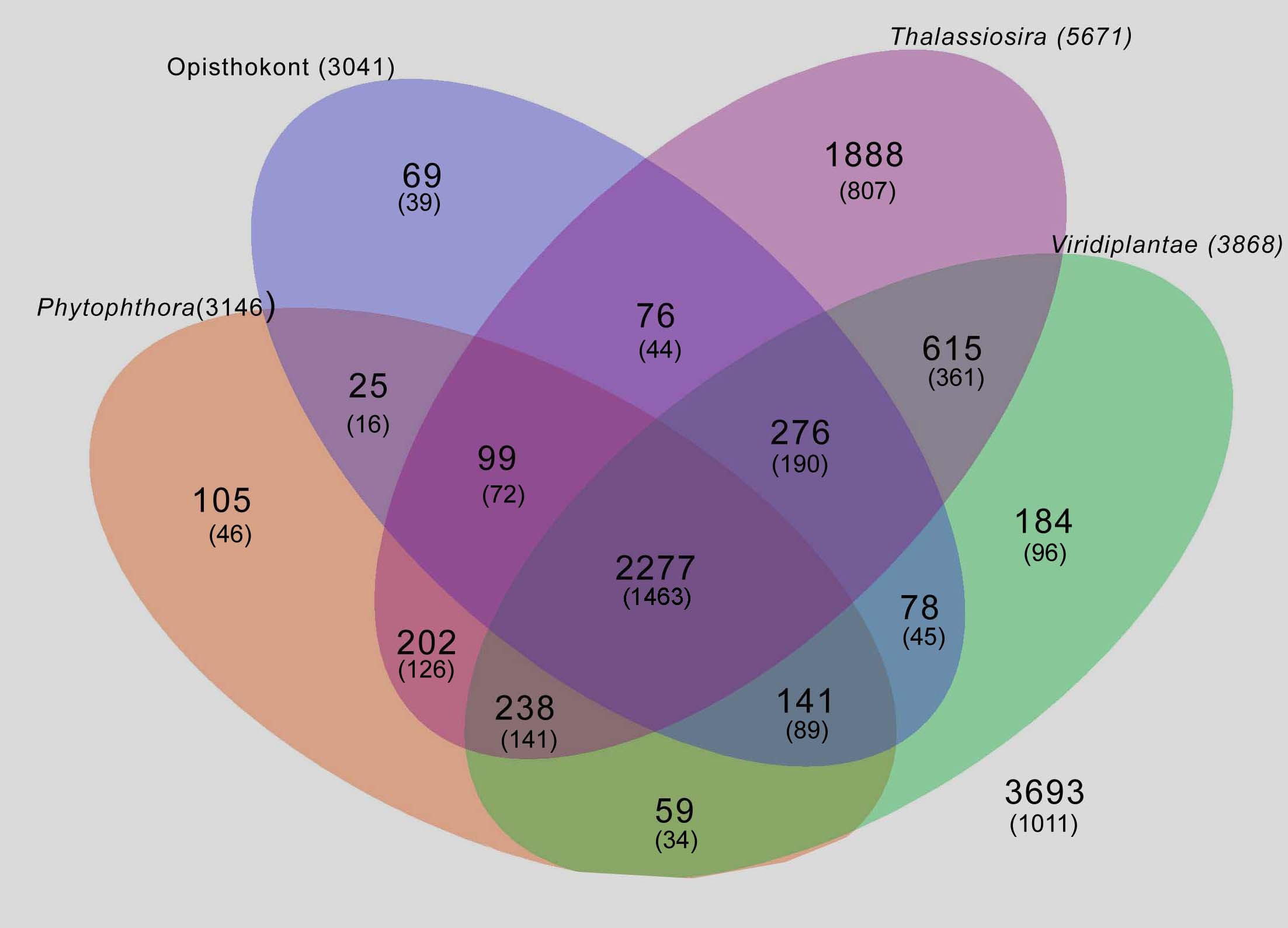

Supplement: Additional file 8 — Supplementary Figure S3. Distribution of P. tricornutum PDFs in other organismal groups. Numbers in parentheses indicate the number of genes with defined protein domains (PDF) and the number outside the parentheses represent the total number of genes in each organismal group. [file gb-2010-11-8-r85-S8.JPEG]

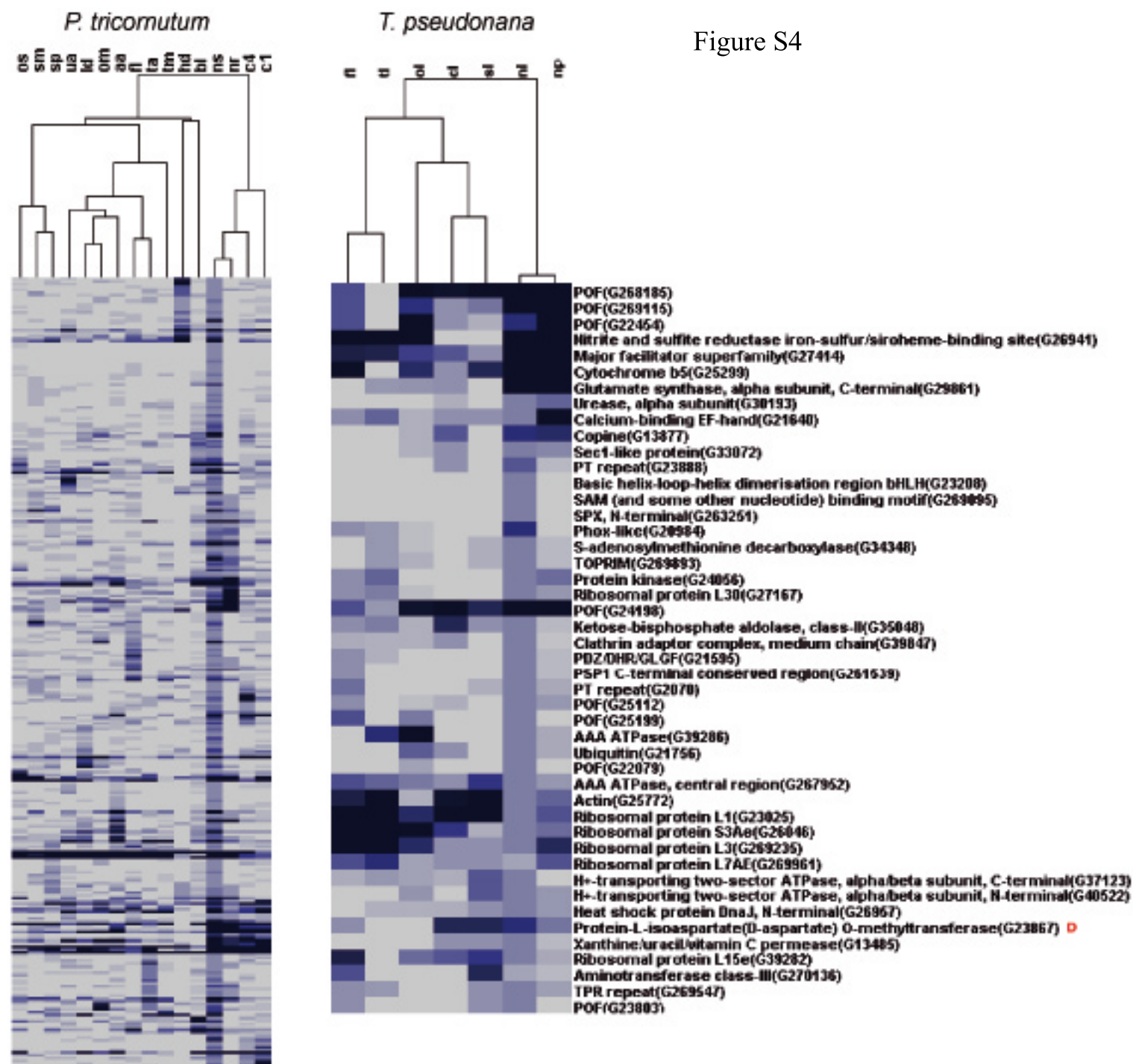

Figure S4

Supplement: Additional file 10 — Supplementary Figure S4. Hierarchical clustering of transcripts defined as being differentially expressed under the nitrate starved condition (NS) in P. tricornutum along with the hierarchical clustering of corresponding orthologs expressed in the nitrate limited condition (NL) in T. pseudonana. Expression levels are shown in an increasing scale from grey to dark blue, and are based on frequencies of ESTs in each library (see Materials and methods). For two-letter library codes, see Table 1 and the Figure 7 legend. [file gb-2010-11-8-r85-S10.PDF]

Figure S5

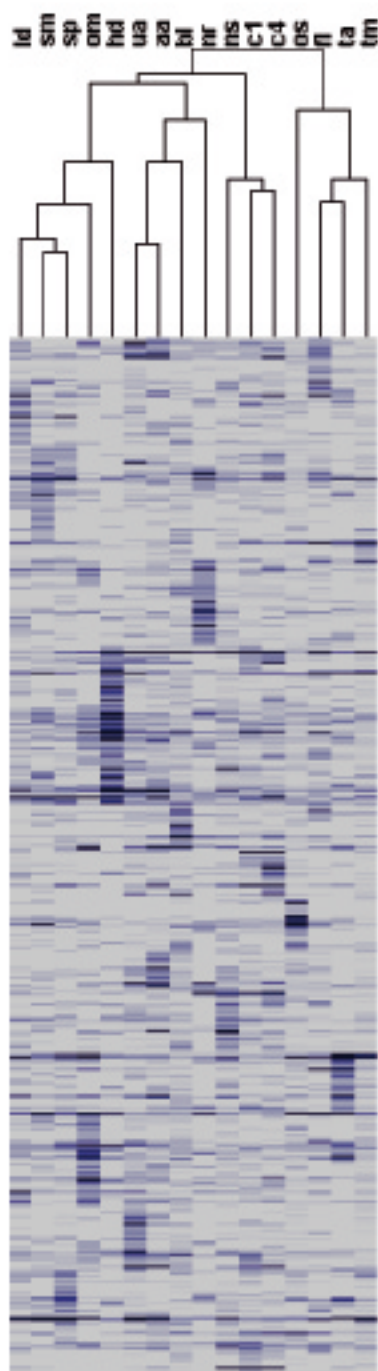

Supplement: Additional file 11 — Supplementary Figure S5. Hierarchical clustering showing the expression patterns of P. tricornutum orthologs of the novel genes identified by tiling array in T. pseudonana [42]. Expression levels are shown in an increasing scale from grey to dark blue, and are based on frequencies of ESTs in each library (see Materials and methods). For two-letter library codes, see Table 1. [file gb-2010-11-8-r85-S11.PDF]

Figure S6

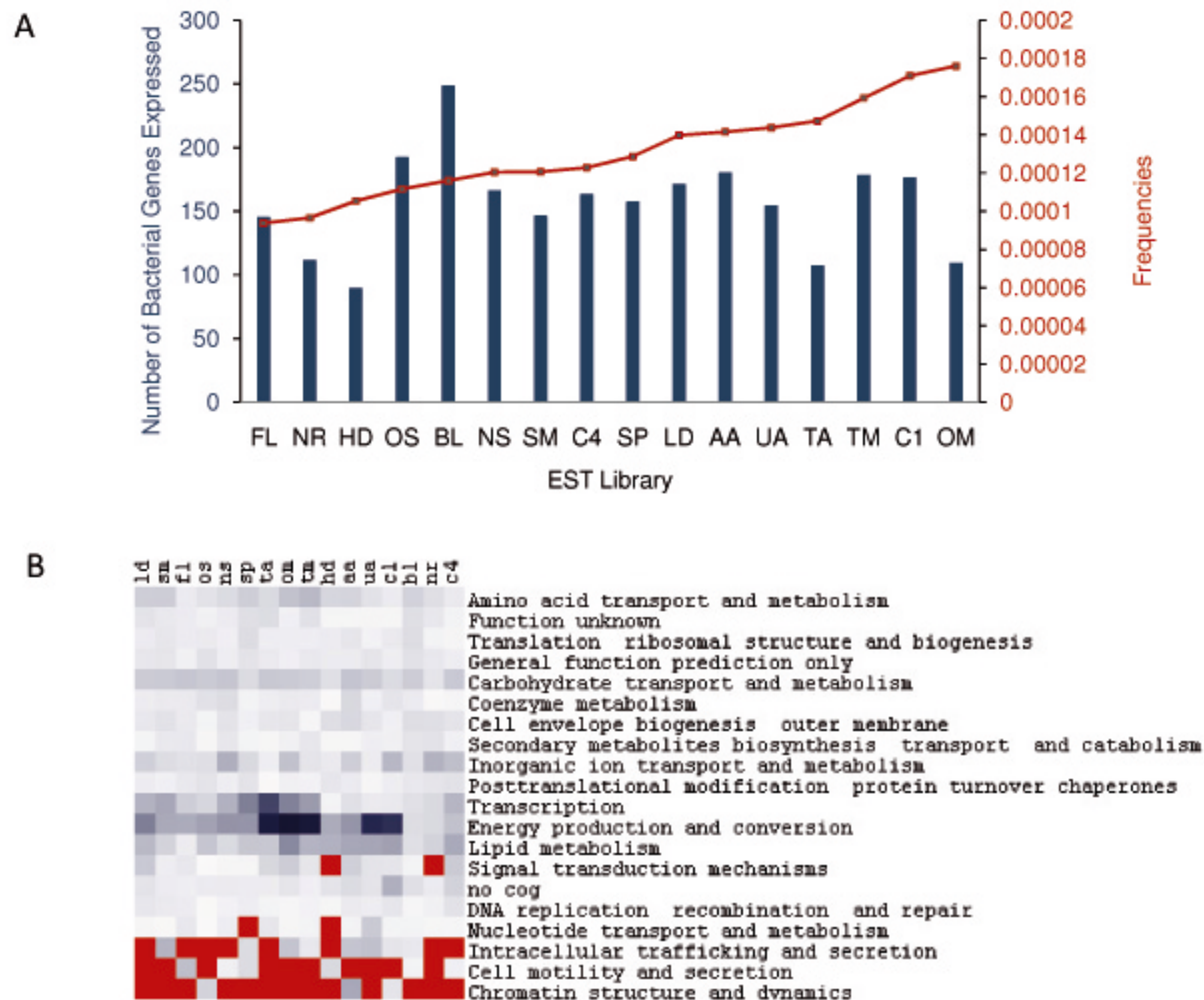

Supplement: Additional file 12 — Supplementary Figure S6. Expression of bacterial orthologous genes in P. tricornutum. (A) Plot showing the number of transcripts of bacterial origin expressed across the 16 different growth conditions. The primary y-axis shows the number of transcripts and the secondary y-axis shows the average frequency of these expressed transcripts. (B) Expression profiling of the genes of putative bacterial origin along with their COG categories. The gradient of blue shows the level of expression, with the darker colors being the highly expressed genes. The red color shows the lack of expressed transcripts. For two-letter library codes, see Table 1. [file gb-2010-11-8-r85-S12.PDF]
